# Staged limb salvage after an infected full-thickness shoulder-girdle flame burn with osteoarticular exposure in an elderly patient: A case report with 3.5-year follow-up

**DOI:** 10.1097/MD.0000000000051338

**Published:** 2026-10-23

**Authors:** Nguyen Ngoc-Huyen, Nguyen The-Hoang, Nguyen Quang-Vinh, Le Hanh

**Affiliations:** a Department of Plastic, Reconstructive Surgery and Upper Extremity Surgery, Institute of Traumatology, Orthopedics, and Plastic Surgery, Central Military Hospital 108, Hanoi, Vietnam; b Department of Orthopedics, Trauma and Plastic Surgery, University Hospital Leipzig, Leipzig, Germany; c Department of Joint Surgery, Institute of Traumatology, Orthopedics, and Plastic Surgery, Central Military Hospital 108, Hanoi, Vietnam.

**Keywords:** case report, flame burn, latissimus dorsi flap, limb salvage, shoulder girdle

## Abstract

**Rationale:**

In extensive shoulder-girdle burns, the critical question may not be flap selection itself, but whether meaningful limb salvage remains justified, particularly in an elderly, frail patient with a delayed infection but preserved distal neurovascular function.

**Patient concerns:**

A 73-year-old woman with diabetes, hypertension, and cachexia (body mass index, 16.9 kg/m^2^) was transferred approximately 3 weeks after a flame burn. She presented with an extensive infected wound extending from the neck base to the distal third of the left arm.

**Diagnoses:**

The burn extended through deep soft tissues to the shoulder capsule, rotator cuff, proximal humerus, scapula, and acromioclavicular and glenohumeral regions, consistent with a fourth-degree injury. Cultures grew *Escherichia coli* and *Acinetobacter baumannii*. No injury to the brachial plexus and major vessels was identified. Sepsis could not be retrospectively classified.

**Interventions:**

Following systemic and nutritional optimization, culture-directed antibiotics, 6 serial debridements, and negative-pressure wound therapy at −125 mm Hg, the infection was clinically controlled. Four weeks after admission, the defect was reconstructed with a 20 × 15 cm ipsilateral pedicled latissimus dorsi myocutaneous flap and skin grafting.

**Outcomes:**

The flap survived completely without significant donor-site complications. At 3.5 years, the wound remained healed without recurrent drainage, ulceration, or further surgery. Active shoulder motion remained markedly limited, whereas passive shoulder motion and distal upper-extremity function were near those of the contralateral side. The patient remained independent in daily activities, with a disabilities of the arm, shoulder and hand score of 28/100.

**Lessons:**

Preserved distal neurovascular function and realistic residual-limb utility may justify staged salvage despite severe shoulder destruction. The favorable outcome reflects careful patient selection, stabilization, debridement, infection control, and durable vascularized coverage rather than the superiority of a specific flap or a general recommendation to delay reconstruction.

## 1. Introduction

Deep burns of the shoulder girdle that expose or destroy bones, joints, tendons, and capsules are uncommon and difficult to reconstruct. Published burn-reconstruction literature largely addresses flap selection, microsurgical timing, or postburn contracture. Reports specifically describing infected shoulder-girdle flame burns with major osteoarticular exposure and long-term limb salvage outcomes remain limited.^[1–8]^ In these situations, the decisive question may be whether the limb still has sufficient residual functional potential to justify salvage before the reconstructive method is chosen.

Therefore, the present case is not presented as a novel use of the pedicled latissimus dorsi (LD) flap. Its clinical interest lies in the sequence of decisions required in a 73-year-old woman who presented late with a deeply infected full-thickness flame burn, substantial shoulder-girdle tissue loss, and marked physiological frailty, but retained distal perfusion, motor function, and sensation of the injured limb. We describe how limb salvage, timing of reconstruction, flap selection, and later shoulder management were individualized, and then report the durability and functional limitations of the result at 3.5 years.

## 2. Case report

All procedures adhered to the guidelines of the Declaration of Helsinki (1964) and its subsequent amendments. Institutional Review Board approval was not required for this single-patient case report, according to our institutional policy. Written informed consent was obtained from the patient for publication of this case report and the accompanying images and video. The paper has been reported in line with the case report checklist.^[9]^

In December 2022, a 73-year-old right-hand-dominant, retired woman with a history of diabetes mellitus and hypertension was referred to our institution approximately 3 weeks after sustaining a flame burn while cooking at home. According to her family, she had suddenly lost consciousness for an unclear reason and fallen into the cooking flame. The duration of flame exposure and whether clothing ignited could not be reliably reconstructed. Initial treatment at a local hospital included fluid resuscitation, antibiotics, analgesia, and routine local wound care. Despite these measures, progressive tissue necrosis, wound infection, and deterioration of her general condition prompted transfer for further management. Before the injury, she was independently mobile and independent in activities of daily living.

On admission, the patient’s height was 150 cm, weight 38 kg, and body mass index 16.9 kg/m^2^. Temperature was 38.5^o^C, heart rate 102 beats/minute, respiratory rate 20 breaths/minute, blood pressure 160/100 mm Hg, and peripheral oxygen saturation was 94%. Laboratory investigations demonstrated marked leukocytosis (20.46 × 10^9^/L) and neutrophils (86.6%), anemia (red blood cell count, 2.93 × 10^12^/L; hematocrit, 25.3%), thrombocytosis (547 × 10^9^/L), lactate 3.8 mmol/L, elevated liver enzymes (aspartate transaminase, 222.0 U/L; alanine aminotransferase, 254.5 U/L), hypoalbuminemia (29.6 g/L), hypoproteinemia (59.3 g/L), urea 8.9 mmol/L, and creatinine 74 μmol/L. Glycated hemoglobin was 7.2%, and hypertension 160/100 mmHg. Blood cultures were negative. A C-reactive protein value was not available in the retrievable record.

The available admission data supported a severely infected wound with systemic physiological compromise but did not allow complete retrospective sequential organ failure assessment scoring because bilirubin, partial pressure of oxygen/fraction of inspired oxygen, Glasgow Coma Scale documentation, and complete vasopressor/hemodynamic data were unavailable. Accordingly, although systemic infection was clinically suspected and treated, we do not label the presentation as sepsis or multiple organ dysfunction in this report under current Sepsis-3 terminology.^[10]^

Local examination revealed an infected soft tissue defect measuring approximately 40 × 25 cm, involving the left shoulder girdle and proximal upper extremity from the base of the neck toward the left elbow. No other cutaneous burn site was documented at our institution. Because the patient was transferred approximately 3 weeks after injury, the presence or absence of an acute inhalation injury could not be reliably determined retrospectively from the available record. The burn was full thickness and extended beyond the skin into deeper tissues, with exposed and infected proximal humerus, scapula surface, glenohumeral joint, acromioclavicular region, and rotator cuff/scapular structures (Fig. 1). This depth was confirmed operatively by the presence of nonviable tissue extending to deep soft tissue and osteoarticular structures, and no histological burn-depth assessment was performed.

**Figure 1.**
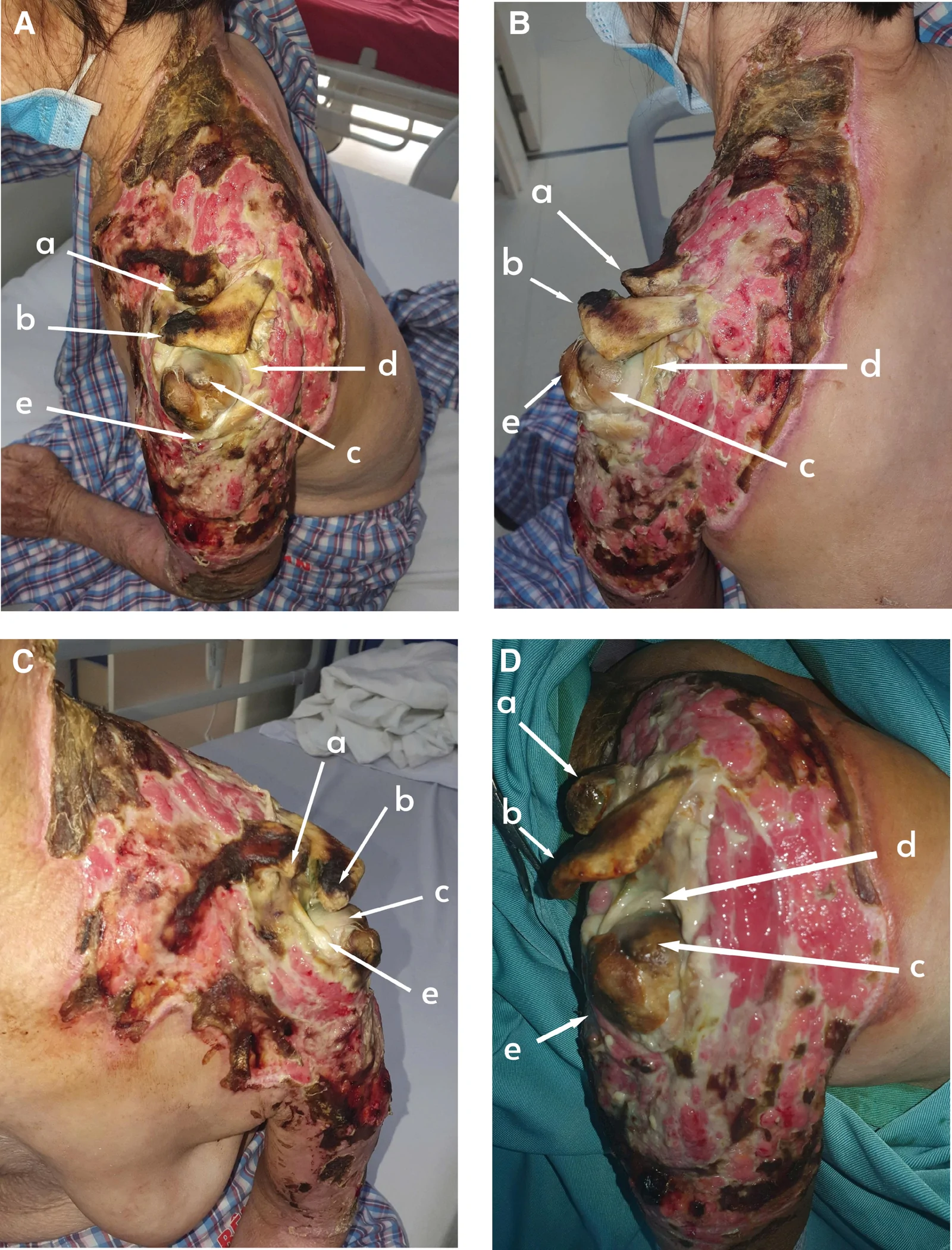
Clinical appearance at presentation, approximately 3 weeks after the flame burn injury. Four views demonstrate an extensive, infected, full-thickness soft tissue defect extending from the base of the neck toward the left elbow, with direct exposure of major shoulder-girdle osteoarticular structures. The lettered arrows identify (a) the distal clavicle, (b) acromion, (c) humeral head, (d) glenoid, and (e) necrotic rotator cuff/capsular tissue. (A) Superior view; (B) superior-posterolateral view; (C) anterolateral view; (D) posterior-oblique view.

The exposed portion of the joint capsule and rotator cuff within the injured zone was necrotic and was removed during debridement, along with other nonviable soft tissue and devitalized portions of involved bone and joint surfaces. The glenoid and a residual portion of the humeral head remained structurally preservable. No injury to the brachial plexus, axillary nerve, or major upper-extremity vessels was identified clinically or intraoperatively. The radial pulse was palpable, and capillary refill in the fingers was normal. Distal light-touch sensation and gross elbow, wrist, and digital motor function were clinically comparable with the contralateral side. Formal Doppler ultrasonography, angiographic perfusion quantification, Medical Research Council (MRC) grading, and quantitative sensory testing were not performed at baseline because distal perfusion and neurological function were clinically intact.

Wound cultures obtained during the debridement period grew *Escherichia coli* and *Acinetobacter* baumannii. The archived record did not reliably distinguish a formal deep-tissue specimen from a wound swab. The isolates were reported susceptible to Cefoperazone/Sulbactam and Tobramycin. The documented culture-directed regimen consisted of intravenous Cefoperazone/Sulbactam 3 g/day combined with intravenous Tobramycin 160 mg/day for 10 days. No bacteremia was demonstrated. Although infection involved exposed bone and joint surfaces, no bone biopsy/histologic criteria for osteomyelitis and no joint aspirate criteria for septic arthritis were available.

Because the patient was physiologically frail and the boundary between viable and nonviable tissue remained incompletely demarcated, immediate definitive reconstruction was deferred. Treatment during the following 4 weeks focused on glycemic and blood pressure control, correction of anemia and hypoproteinemia as clinically indicated, and nutritional support under the hospital nutrition service with a target intake of at least 2000 kcal/day, but a formal protein target was not retrievable. Six debridement procedures were documented: 1 major operating-room debridement addressing devitalized bone/joint tissue and 5 additional bedside debridements of residual nonviable soft tissue. Negative-pressure wound therapy (NPWT) (V.A.C. Ulta Therapy System; Solventum, formerly 3M Health Care/KCI) was used at − 125 mm Hg in continuous mode between debridements until definitive reconstruction, with dressing changes every 3 to 5 days.

Readiness for reconstruction was based on converging clinical findings rather than microbiological sterility, including the patient’s general condition and comorbidities had stabilized, no further progressive necrosis was present, the wound showed healthy granulation and viable bleeding tissue after debridement, and the infection was considered clinically controlled. Flap coverage did not require a negative repeat culture as a mandatory requirement. Following final debridement, the residual defect measured approximately 20 × 18 cm and continued to expose the glenohumeral/acromioclavicular region, proximal humerus, scapula, and rotator cuff structures (Fig. 2A).

**Figure 2.**
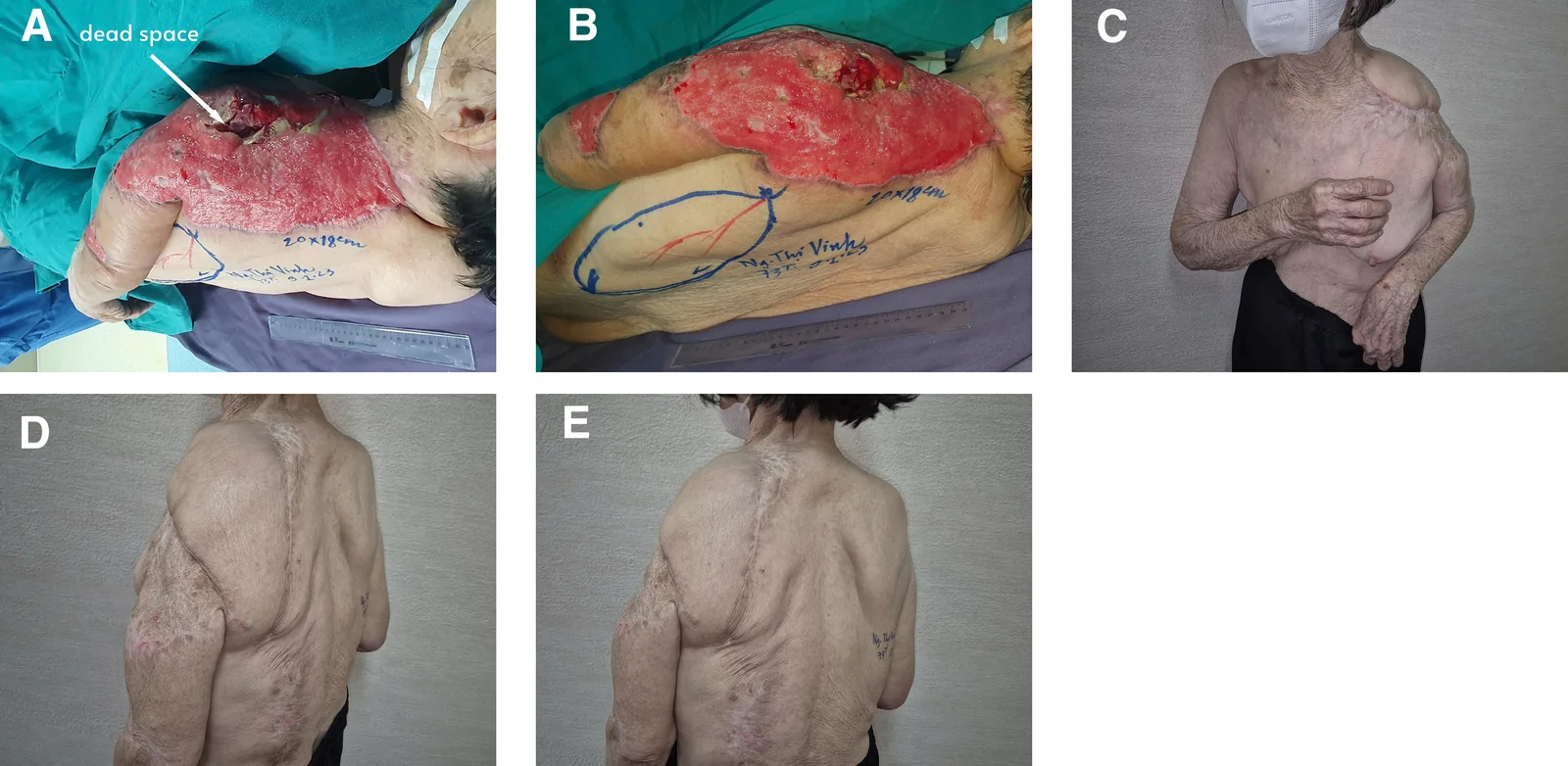
Wound preparation, flap planning, and long-term appearance. (A) Wound approximately 4 weeks after admission following serial debridement and negative-pressure wound therapy, showing the residual deep cavity/dead space in the shoulder region. (B) Preoperative design of the ipsilateral pedicled LD myocutaneous flap. (C–E) Clinical appearance at the 3.5-year follow-up from anterior, posterolateral, and posterior views, demonstrating durable coverage of the reconstructed shoulder region and a healed donor-site scar without clinically apparent seroma or wound breakdown.

An ipsilateral pedicled LD myocutaneous flap of approximately 20 × 15 cm was selected (Fig. 2B). The thoracodorsal bundle was identified and protected during flap elevation, and flap perfusion was confirmed clinically after elevation. No preoperative angiography of the thoracodorsal pedicle was performed. The flap was rotated approximately 180^o^ into the shoulder defect. The muscle component was inset deeply to obliterate the residual cavity created after removal of nonviable shoulder structures, while the skin paddle covered the principal exposed region. Residual superficial defects were covered with split-thickness skin grafts harvested from the groin. The donor site was closed primarily. Operative time was 160 minutes, estimated blood loss was 200 mL, and 1 unit of packed red blood cells was transfused.

Postoperatively, the left upper limb was supported in a Desault-type sling. A rubber drain was placed at the operative site, and flap viability was monitored clinically through a dressing window by skin color, temperature, capillary refill, and general appearance. The skin graft took satisfactorily. The flap remained fully viable, no donor-site seroma or other clinically significant donor-site complication occurred. The wounds healed without further surgical intervention, and the patient was discharged 2 weeks after reconstruction.

From postoperative day 3, the patient began supervised therapy with active elbow, wrist, and finger motion and gentle passive mobilization as permitted. After wound healing, outpatient therapy emphasized elbow, wrist, and hand motion together with passive shoulder flexion/extension, abduction/adduction, and internal/external rotation. After 4 weeks, she continued the same program at a local rehabilitation facility. The Desault support was removed for exercises and reapplied during rest and at night for approximately 3 months. Follow-up examinations were performed at approximately 3- to 6-month intervals, although standardized functional instruments were not administered serially.

At the 3.5-year follow-up, the reconstructed shoulder remained healed without pain, recurrent infection, wound breakdown, chronic drainage, ulceration, or need for further surgical intervention (Figs. 2C–2E). No clinically apparent donor-site seroma, problematic scar, or new donor-site complaint was reported. Active shoulder excursion was markedly limited and was estimated clinically at approximately 5 to 10% of the contralateral side, but passive shoulder motion was near the contralateral side. Elbow and wrist range of motion, distal motor function, light-touch sensation, and grip in routine clinical testing were broadly comparable with the contralateral limb, but formal goniometry, dynamometry, and MRC grading were not obtained. The patient could use the reconstructed limb independently for routine activities of daily living without pain or subjective weakness (Figs. 3A –3C). The full Disabilities of the Arm, Shoulder and Hand questionnaire was administered once at the 3.5-year clinic visit and was not retrospectively reconstructed from the medical record. The score was 28/100.^[11]^ The questionnaire was explained to the patient in her mother language as needed, but the version used in this case was not documented as a formally validated mother language translation. Three-dimensional computed tomography demonstrated the residual post-debridement shoulder-girdle skeletal architecture and persistent congruence of the residual humeral head with the glenoid (Figs. 3D–3F). Because only volume-rendered images are presented, these images were not used alone to exclude subtle osteolysis or osteomyelitis.

**Figure 3.**
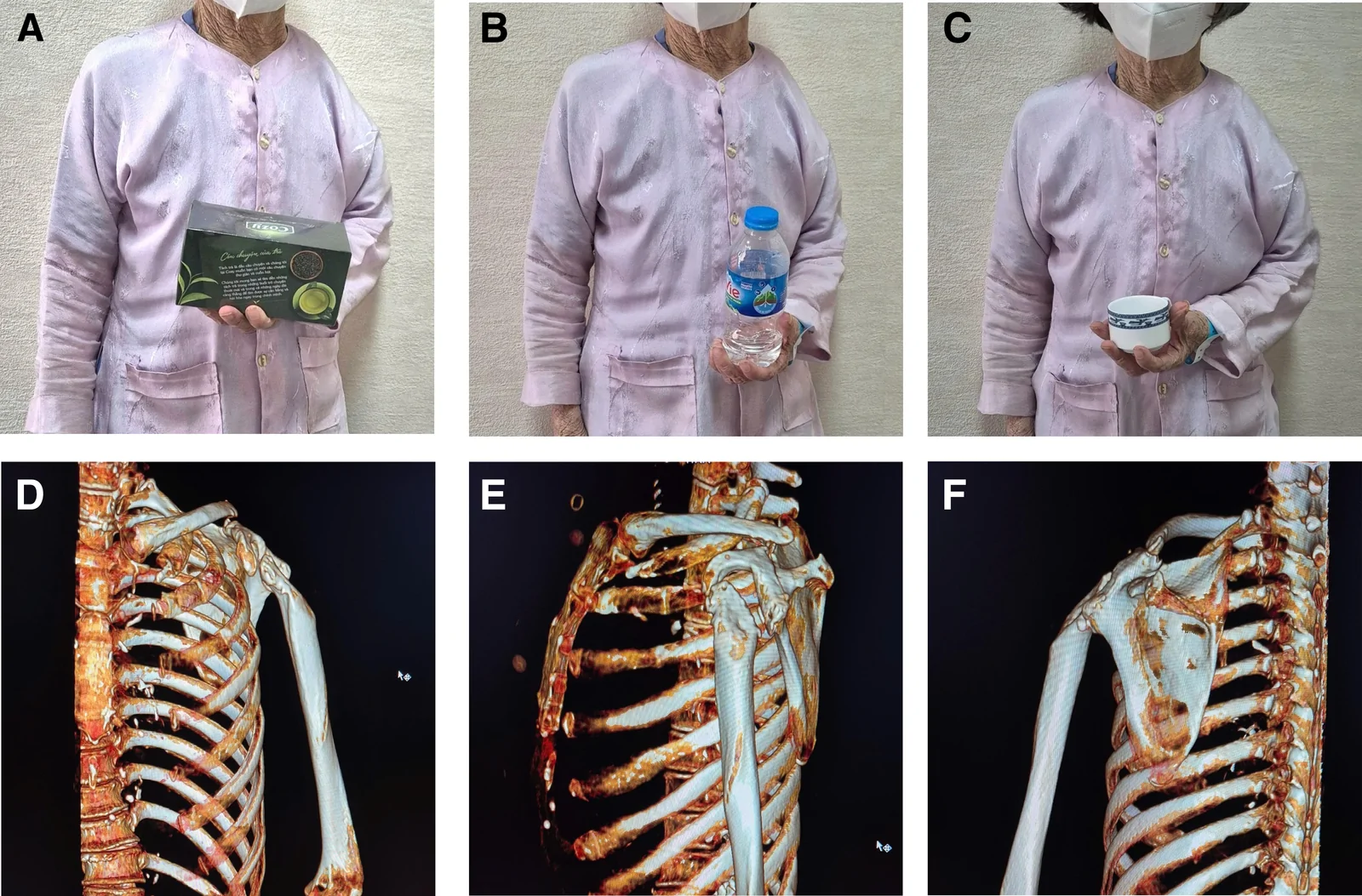
Functional and radiological appearance at the 3.5-year follow-up. (A–C) The patient demonstrates independent use of the reconstructed left upper extremity while holding a box, a water bottle, and a cup, illustrating functional use of the elbow, wrist, and hand despite marked limitation of active shoulder motion. (D–F) Three-dimensional computed tomography reconstructions showing the residual post-debridement shoulder-girdle skeletal architecture and the relationship of the residual humeral head to the glenoid. These volume-rendered images are descriptive and are not used alone to exclude subtle osteolysis or osteomyelitis.

At final follow-up, the patient reported that she was very satisfied with preservation of the left upper limb. She considered the marked limitation of shoulder motion acceptable because the arm was painless, the elbow, wrist, and hand remained useful, and she could perform her usual daily activities independently. She had not requested additional reconstructive or stabilizing surgery. The patient’s clinical timeline is presented in Table 1.

**Table 1:** Clinical timeline of the patient.

Time	Key clinical event
3 weeks before admission	Flame burn while cooking after transient loss of consciousness; duration of exposure unknown. Initial treatment at a regional hospital.
December 2022 (admission)	Transfer with extensive infected full-thickness shoulder-girdle wound, cachexia, leukocytosis, hyperlactatemia, anemia, hypoalbuminemia, and preserved distal perfusion/neurological function.
Hospital weeks 0–4	Medical optimization, nutrition, culture-directed antimicrobial therapy, 1 major operative and 5 bedside debridements, and continuous NPWT at −125 mm Hg.
4 weeks after admission	Final debridement and definitive reconstruction with pedicled LD myocutaneous flap plus STSG.
Postoperative day 3 onward	Progressive passive and active mobilization of the elbow, wrist, and hand with supervised rehabilitation.
2 weeks after reconstruction	Wounds healed; discharge with outpatient rehabilitation.
Up to 3 months after reconstruction	Desault support used during rest/night; removed for rehabilitation.
Every 3–6 months	Clinical follow-up; no recurrent wound breakdown or additional surgery documented.
3.5 years	Final comprehensive review, functional assessment, DASH 28/100, clinical photography, and CT assessment.

CT = computed tomography, DASH = disabilities of the arm, shoulder and hand, LD = latissimus dorsi, NPWT = negative-pressure wound therapy, STSG = split-thickness skin graft.

## 3. Discussion

### 3.1. What was the actual limb salvage decision?

The reconstructive technique in the present case was conventional. The principal clinical question was whether a limb with dramatic local shoulder destruction still retained enough functional value to justify salvage. At admission, options included proximal amputation (shoulder disarticulation or, if more extensive resection were required, forequarter amputation), staged salvage, or immediate definitive reconstruction. Major proximal amputation can control an otherwise unsalvageable injury or infection. However, it produces profound functional loss, and it is itself a major operation.^[12]^ In this patient, the hand and forearm were well perfused, distal motor and sensory function were preserved, the major neurovascular axis was clinically intact, and the local infection appeared potentially controllable. These findings favored an attempt at salvage rather than removal of an otherwise viable and potentially useful distal limb.

As a result, this distinction between local structural severity and residual-limb utility is central to the report. Extensive osteoarticular exposure did not necessarily indicate that the entire upper extremity was functionally unsalvageable. However, pursuing limb salvage was neither an obvious choice nor guaranteed to succeed. If distal perfusion had been compromised, brachial plexus function had been lost, infection had remained uncontrollable, or the patient’s physiology had failed to improve, the balance could reasonably have shifted toward amputation. Therefore, Figure 4 describes this case as a conceptual pathway and should not be interpreted as a validated algorithm.

**Figure 4.**
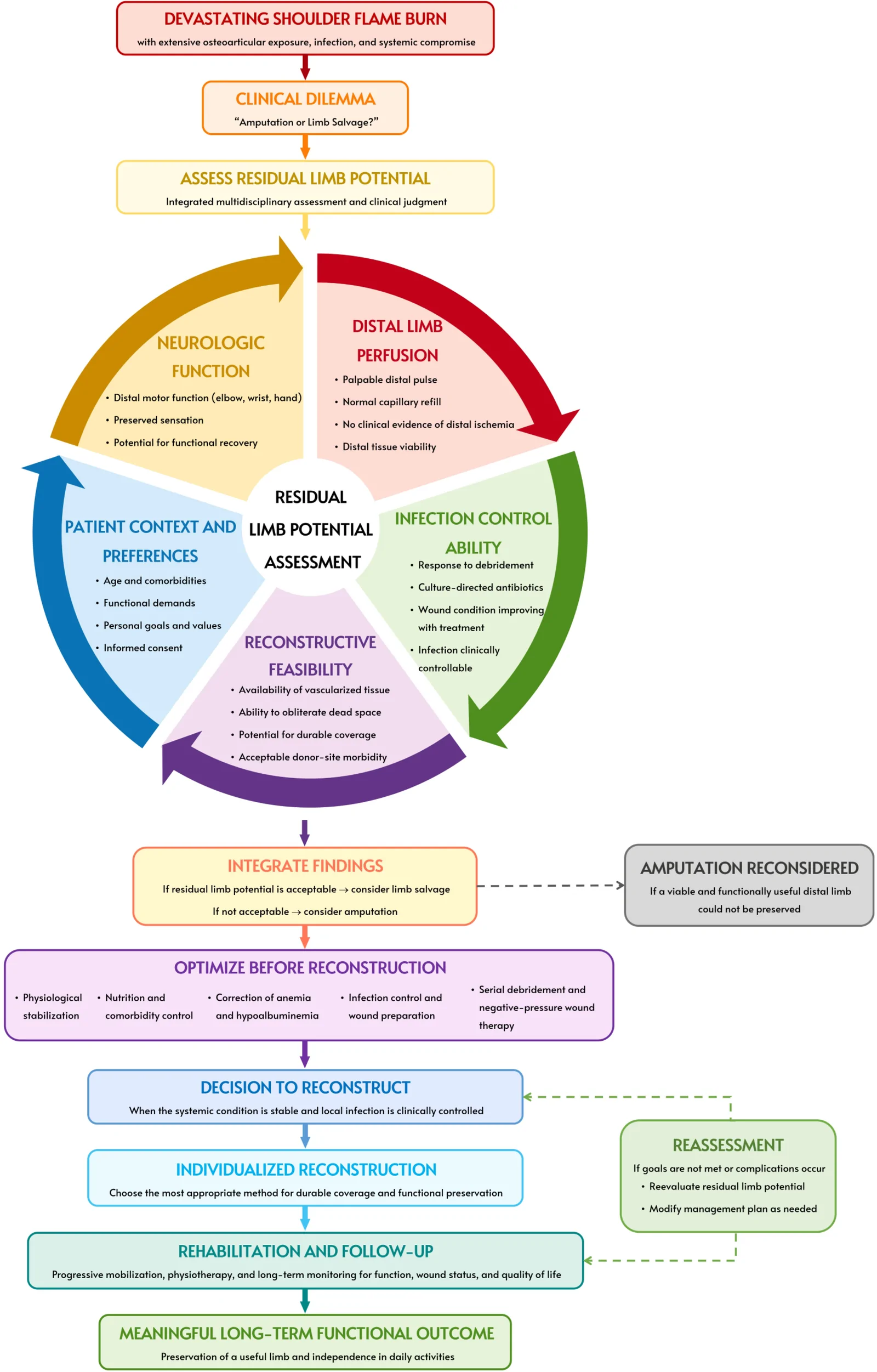
Case-specific conceptual map of the clinical reasoning used in this patient. The figure summarizes the factors considered when balancing amputation against limb salvage, including distal neurological function and perfusion, ability to control infection, reconstructive feasibility, systemic recovery, and patient context. It is intended to illustrate the decisions made in this case rather than to propose a validated treatment algorithm.

### 3.2. Why was reconstruction staged rather than immediate?

At transfer, the patient was malnourished and anemic, with an actively infected wound and an incompletely demarcated zone of necrosis. Immediate flap coverage was considered inappropriate because residual nonviable tissue might still have been present. Moreover, the patient remained medically fragile, and further assessment of the wound and overall clinical condition was required before definitive reconstruction. For this reason, the initial phase prioritized resuscitative and medical care, nutritional support, repeated debridement, antimicrobial therapy, and NPWT. NPWT was used only as an adjunct between debridements, and it did not substitute for removal of devitalized tissue or infection control.

The first 4-week interval of the staged approach should be understood as case-specific wound preparation rather than evidence that delayed flap coverage is generally preferable. Burn-reconstruction literature supports individualized timing based on wound biology, infection, and patient physiology.^[2–5]^ In this case, the staged sequence was associated with a durable result, but causality cannot be assigned to the delay itself.

### 3.3. Why was a pedicled LD flap used?

Once definitive reconstruction became appropriate, the available options included local or regional fasciocutaneous/perforator flaps,^[6,13–16]^ trapezius- or pectoralis-major–based flaps,^[7,16,17]^ pedicled LD tissue,^[18,19]^ and free tissue transfer.^[2–5,8,14]^ Smaller regional flaps were considered insufficient for the combined requirements of broad surface coverage and obliteration of a deep shoulder cavity. Trapezius- and pectoralis-major–based options can be useful for selected shoulder-girdle defects, but they provide different arcs of rotation, surface areas, and tissue volumes.^[13,16,17]^ Free tissue transfer remained technically feasible, but it would have required recipient-vessel planning. Besides, it is also a more complex procedure in a patient who had only recently recovered from systemic illness than coverage by a pedicle flap.^[2,4,14]^

The ipsilateral pedicled LD flap was selected because it offered a large volume of well-vascularized muscle, a long and reliable pedicle, broad reach to the shoulder, rapid inset, and the ability to fill dead space.^[18,19]^ The choice should not be interpreted as evidence that LD is superior to other flaps. Rather, it matched this patient’s defect and physiological constraints.

Shoulder morbidity from LD flap harvest is also a major concern. Systematic review data show that loss of the LD muscle can reduce shoulder strength, particularly extension, adduction, and internal rotation, and some patients report donor-site discomfort.^[20]^ However, in this patient, normal shoulder mechanics had already been profoundly disrupted by necrosis and debridement of the capsule, rotator cuff, and osteoarticular structures. No donor-site seroma or clinically apparent new distal upper-extremity weakness was observed, but quantitative pre- and postoperative shoulder strength testing was not available. Therefore, the contribution of LD flap harvest to the final shoulder limitation cannot be separated from the effects of the original injury and required debridement.

### 3.4. Shoulder stabilization and long-term functional meaning

Immediate arthrodesis was not considered appropriate because the infection remained active and tissue necrosis was still evolving. Moreover, arthrodesis would have required placement of fixation implants in an infected surgical field and, in such circumstances, would still have needed to be combined with well-vascularized soft tissue coverage to ensure successful arthrodesis. Temporary Desault support was only used during the early healing period. No later arthrodesis was performed. At long-term follow-up, the shoulder remained markedly limited but painless, the residual humeral head remained congruent with the glenoid, and the patient considered distal limb utility and daily independence satisfactory. The principal functional success was preserving a viable and useful elbow-forearm-hand unit, not restoring normal shoulder function.

### 3.5. Limitations

This report acknowledges some limitations. It describes a single selected patient and cannot establish indications for salvage, optimal timing of reconstruction, or comparative superiority of the flap. Baseline and serial standardized functional scores were unavailable, and goniometric shoulder/elbow/wrist measurements, dynamometric grip testing, and formal MRC grading were not obtained. The disabilities of the arm, shoulder and hand score was measured only once at the 3.5-year timepoint, and the version that the patient used was not documented as a formally validated Vietnamese translation. Complete data required for retrospective sequential organ failure assessment scoring were lacking, so the manuscript avoids a formal Sepsis-3 or multiple organ-dysfunction diagnosis. The microbiology record did not permit definitive classification of the wound specimen as a deep-tissue culture, and formal bone biopsy or joint-aspiration criteria for osteomyelitis/septic arthritis were unavailable. Finally, because the original injury destroyed major shoulder stabilizers, the specific contribution of LD flap harvest to long-term shoulder weakness cannot be quantified. Patient selection, especially preserved distal neurovascular function, may have contributed substantially to the favorable limb-preservation outcome.

## 4. Conclusion

This case illustrates that even a severely infected, full-thickness shoulder-girdle flame injury with exposed bone and joint may make limb salvage possible when distal perfusion, neurological function, and useful elbow-forearm-hand function are preserved. In this patient, staged multidisciplinary management followed by pedicled LD coverage was associated with durable wound healing and preservation of an independently useful distal upper extremity despite permanent major loss of shoulder motion. The observations are illustrative rather than prescriptive and should not be interpreted as evidence that delayed reconstruction or the LD flap should be routinely preferred.

## Author contributions

**Conceptualization:** Nguyen Ngoc-Huyen, Nguyen The-Hoang.

**Data curation:** Nguyen Ngoc-Huyen, Nguyen The-Hoang.

**Formal analysis:** Nguyen Ngoc-Huyen, Nguyen Quang-Vinh, Le Hanh.

**Investigation:** Nguyen Ngoc-Huyen, Nguyen The-Hoang, Nguyen Quang-Vinh, Le Hanh.

**Methodology:** Nguyen Ngoc-Huyen, Nguyen Quang-Vinh, Le Hanh.

**Project administration:** Nguyen The-Hoang.

**Resources:** Nguyen Ngoc-Huyen, Nguyen Quang-Vinh, Le Hanh.

**Software:** Nguyen Quang-Vinh, Le Hanh.

**Supervision:** Nguyen The-Hoang.

**Visualization:** Nguyen The-Hoang, Nguyen Quang-Vinh.

**Validation:** Nguyen Ngoc-Huyen.

**Writing – original draft:** Nguyen Ngoc-Huyen, Nguyen The-Hoang.

**Writing – review & editing:** Nguyen Ngoc-Huyen, Nguyen The-Hoang, Nguyen Quang-Vinh, Le Hanh.
